# A qualitative study of discourses on heterosexual anal sexual practice among key, and general populations in Tanzania: implications for HIV prevention

**DOI:** 10.1186/s12889-015-1768-4

**Published:** 2015-04-24

**Authors:** Joyce Wamoyi, Aika Mongi, Mtenga Sally, Deodatus Kakoko, Donat Shamba, Eveline Geubbels, Saidi Kapiga

**Affiliations:** National Institute for Medical Research, Department of Sexual and Reproductive Health, P.O Box 1462, Mwanza, Tanzania; Mwanza Intervention Trial Unit (MITU), Mwanza, Tanzania; Ifakara Health Institute (IHI), Impact Evaluation Thematic Group, Dar es Salaam, Tanzania; Department of Behavioural Sciences, Muhimbili University of Health and Allied Science (MUHAS), School of Public Health, Dares Salaam, Tanzania

**Keywords:** Heterosexual anal sex, Discourses, Key populations, General populations, HIV, Tanzania

## Abstract

**Background:**

The risk of contracting HIV through heterosexual anal sex (HAS) is significantly higher than from vaginal intercourse. Little has been done to understand the discourses around HAS and terms people use to describe the practice in Tanzania. A better understanding of discourses on HAS would offer useful insights for measurement of the practice as well as designing appropriate interventions to minimise the risks inherent in the practice.

**Methods:**

This study employed qualitative approaches involving 24 focus group discussions and 81 in-depth interviews. The study was conducted in 4 regions of Tanzania, and included samples from the general population and among key population groups (fishermen, truck drivers, sex workers, food and recreational facilities workers). Discourse analysis was conducted with the aid of NVIVO versions 8 and 10 software.

**Results:**

Six discourses were delineated in relation to how people talked about HAS. Secrecy versus openness discourse describes the terms used when talking about HAS. “Other” discourse involved participants’ perception of HAS as something practiced by others unrelated to them and outside their communities. Acceptability/trendiness discourse: young women described HAS as something trendy and increasingly gaining acceptability in their communities. Materiality discourse: describes HAS as a practice that was more profitable than vaginal sex. Masculinity discourse involved discussions on men proving their manhood by engaging in HAS especially when women initiated the practice. Masculine attitudes were also reflected in how men described the practice using a language that would be considered crude. Public health discourse: describes HAS as riskier for HIV infection than vaginal sex. The reported use of condoms was low due to the perceptions that condoms were unsuitable for anal sex, but also perceptions among some participants that anal sex was safer than vaginal sex.

**Conclusion:**

Discourses among young women and adult men across the study populations were supportive of HAS. These findings provide useful insights in understanding how different population groups talked about HAS and offer a range of terms that interventions and further research on magnitude of HAS could draw on when addressing health risks of HAS among different study populations.

## Background

Despite evidence of declining HIV incidence in many parts of the world and increasing access to life-saving antiretroviral drugs (ART), HIV infection remains a major public health problem in sub-Saharan Africa (SSA) where 70% of all new infections occur [1]. Although some recent declines in national HIV prevalence among adults have been reported in Tanzania, with the overall prevalence falling from 7% to 5.1% between 2003 and 2011-12 [2], there is evidence indicating that new infections continue to occur in both key and general populations [1]. Thus, expansion of ART services and strengthening of HIV prevention efforts is a major priority in Tanzania. Effective prevention efforts help to reduce the number of new HIV infections and the pool of HIV-infected patients who will need treatment in the future.

Sexual intercourse is the main mode of HIV transmission among adults globally and sexual behaviour change, including use of condoms, is a key strategy to stem the spread of new infections [3]. Penile-anal intercourse is typically associated with male homosexual relationships, with relatively little attention given to the issues related to anal intercourse in heterosexual relationships [4,5]. Recent studies show that women’s risk of contracting HIV through heterosexual anal sex (HAS) is significantly higher than from vaginal intercourse [6,7]. Receptive anal intercourse has also been associated with increased risk of other STIs, and complications related to these infections, anal cancers among women [8], and changes in vaginal microflora and reproductive tract infections [9,10].

Comprehensive information about the extent to which HAS is practiced in various parts of Tanzania is lacking. Due to the sensitivity of the subject, it is important to first understand the discourses on HAS before implementing a large-scale survey to determine the magnitude of the practice. It is also important to know common terms used to describe the practice in different populations or cultural settings.

Discourses reflect how people think about topics, shape their thinking and may promote particular kinds of behaviour [11]. Dominant discourses condition and govern the ways in which knowledge about sexual health (in our case HAS practice) can (and cannot) be discussed [12]. Hence, by exploring people’s discourses about HAS, we can better understand how their discussions of sexual health and the practice of HAS in particular, are discursively constituted, constructed and changed [11,13]. To date there is little empirical and theoretical literature examining discourses on HAS among key and general populations in countries severely affected by the HIV epidemic. A better understanding of how people talk about the practice of HAS would offer useful insights, contribute on better measures of the practice and inform development of interventions to make the practice safer.

## Methods

### Design

This study employed ethnographic research design involving 81 in-depth interviews (IDIs) and 24 focus group discussions (FGDs). A combination of IDIs and FGDs were used to explore complex issues related to sexual behaviour among the general population as well as key populations, and to obtain a more detailed understanding of how people talked about HAS.

### Study sites and population

The study was implemented from July until October 2012 at four sites in Dar es Salaam, Morogoro, Mwanza and Tanga regions as a collaborative effort of key Tanzanian institutions. Given that the primary goal of the study was to examine HAS within the context of increased risk of HIV infection, we included participants of reproductive age (15-49 years old) from the general population as well as from key populations based on WHO categorization [14]. This included men and women from the general population; women working in food and recreational facilities (FRFs); female sex workers (FSWs) working in brothels and streets; male truck drivers; and fishermen working on Lake Victoria. Sex workers [15-19], women working in FRFs [20-24], truck drivers [25,26] and fishermen [27,28] are known to be key populations at increased risk of HIV and other STIs. We included a wide age range in order to obtain views from young people and adults.

### Description of the data collection methods

Data collection commenced with FGDs which were instrumental in initiating the discussions in relation to how people talk about HAS, and the meanings they attach to the practice. Twelve FGDs were conducted with key populations and 12 with the general population . The questions were limited to general issues and no personal experiences were discussed. After each FGDs, 2-4 participants were selected to participate in IDIs in order to explore personal experiences and hence, differentiate the normative views as presented in FGDs to the real practice of individuals. A total of 81 IDIs were conducted with the following: 8 truck drivers (Morogoro region); 9 women working in FRFs and 8 fishermen from Mwanza region; 8 female sex workers from Dar Es Salaam; 16 rural general population and 16 urban general population from Tanga region; and 16 rural general population from Morogoro region. We selected IDI participants to ensure variation by responses given during group discussions and to ensure that personal experiences were captured.

Both the FGDs and IDIs were conducted using a semi-structured guide to explore a range of topics, including sexual practices, context within which HAS is practiced, reasons for engaging in HAS, meaning and discourses around HAS, perceptions and attitudes of HAS, perceived link between HAS and HIV and protection use during HAS. All the IDIs and FGDs were conducted with a researcher of the same sex in a private location and were audio recorded, with the exception of FSWs interviews.

### Recruitment

The truck drivers were recruited from four truck stops along a major highway in Morogoro region. Fishermen were recruited by visiting the fishing communities on the shores of Lake Victoria where we selected two communities based on their accessibility, type of fishing and residential composition (dominated by migrants versus indigenous communities). For both the fishermen and truck drivers, snow ball sampling was employed. Initially, 2-3 individuals were approached for each of these populations and asked to invite their colleagues to participate in FGDs and a sample in IDIs. Women employed in FRFs in Mwanza city were recruited after consultation with the facility managers in a neighbourhood with a high concentration of facilities. FSWs in Dar es Salaam were recruited after identifying brothels and streets where they hanged-out for clients. The owners of the brothels were identified and consulted and eventually assisted in the identification of initial FSWs and thereafter snowball sampling was used to identify subsequent FSWs from the brothel and the streets. Participants from the general population in Morogoro and Tanga regions were randomly recruited at hamlet level after community meetings.

### Data processing and analysis

Following data collection, tapes were transcribed verbatim at each research site. In order to maintain quality and richness of content, we analysed data in Kiswahili (local language used in IDIs and FGDs) and English translation was not done. Data were analysed in three stages. In the first stage, researchers read through the IDI and FGD transcripts and developed broad codes used to code 10 paper transcripts (4 FGDs, 6 IDIs). These codes were both a priori as well as grounded in the data. In the second stage, finer codes were developed from further reading of the transcripts and discussions among the researchers across the collaborating institutions. All data collected was entered in NVIVO 8 and 10 software for coding.

In the third stage, we examined the individual codes for emerging patterns with regards to the connection between concepts related to HAS, and how participants talked about HAS when with peers, and sexual partners. The analysis was based on the assumption that participants from the general as well as key populations were both influenced by discourses and engaged in shaping and reproducing them. The transcripts were analysed to explore subverting and contesting discourses by asking: what dominant discourses are employed by young people versus adults, general population versus key population, females versus men, when talking about HAS with peers, and sex partners?, and what are the specific ways that participants either construct, or resist and challenge, dominant HAS discourses? Theories were formulated, such as: ‘the way key populations and young people talked about HAS was encouraging the practice’. In order to test this theory, ‘child codes’ and ‘parent codes’ relating to sex workers, fishermen, truck drivers’ and young people’s views on HAS, HAS and assessment of risk were searched, summarised and compared.

Quotations illustrating the main findings were identified. In the presentation of the quotes, ‘I’ refers to the interviewer while ‘R’ is the respondent.

### Ethical considerations

Potential study participants received detailed information about the study and were given opportunity to discuss any issues or concerns before joining the study. The information about the study was provided in Kiswahili using consent forms approved by the Ifakara Health Institute review board and the Medical Research Coordinating Committee of Tanzania National Institute for Medical Research. Written consent was obtained from participants who agreed to join the study after the study information was provided.

It is a standard ethical practice to seek consent of parents or legal guardians for people aged less than 18 years before they join the study. We only sought consent of parents or legal guardians for participants less than 18 years from the general population. Among key population groups, such as FSWs and women working in FRFs, a waiver on parental consent was sought because such women were considered “independent minors” and in most cases, it was not possible to identify their parents or legal guardians in the areas where they were recruited for this study.

## Results

### Socio-demographic characteristics of participants

Most participants were aged 20-30 years and reported to be Christians. The main economic activity among participants from the general population was subsistence farming and petty trade. Most FSW and women working in FRFs had some formal education ranging from having completed primary school to a few having college training. More than half of the women working in FRFs reported having some secondary school education while only one fisherman out of the eight who participated in the IDIs had some secondary school education. Majority of the female participants reported they were single, while most men reported they were married.

### Discourses about HAS

#### Secrecy versus openness discourse

This discourse describes how open versus discrete participants were when talking about HAS and the terms they used to describe the practice. A number of terms were used to describe HAS (Table 1). There were four literal and nine non-literal terms that most participants used to describe HAS. Majority of the participants described HAS using metaphors in order to shroud the practice. These terms were similar among men and women in both the general and key populations.

**Table 1:** **Examples of ms used were described as:**

***Swahili term/metaphor***	**English translation**	**Possible meanings to the metaphors**
**Discrete terms literally referring to anal sex**		
*Kufira*	Sodomy	Anal copulation with a member of the same or opposite sex
K*utomba kwenye mkundu*	To fuck the anus	Sexual penetration through the anal route
*Kumtia nyuma*	To penetrate from the back	Literally to ‘fuck’ through the anal route
*Msenge*	Men-who have sex with men (MSM)	Linking the act of HAS to MSMs
**Terms not literally referring to HAS**		
*Tigo, Zain, Voda*	Names of telephone companies in Tanzania	Symbols of *Tigo, Zain* and *Vodacom* telephone companies are circular in shape resembling an anus
*Shamba dogo*	Small garden	Anal route considered smaller than vaginal route
*Chuma mboga*	Pluck vegetables from the garden	Bending posture which exposes the back area of the body
*Salimia jirani*	Greet neighbour	Anal route considered neighbouring the vaginal route
*Okota mia*	To pick 100 shillings (usually a coin in TZ currency)	The act of bending to pick something
*Kuzama*	To drown	Implies the act of the penis ‘drowning’ in the anal route
*Geuza samaki*	To turn the fish	A metaphor used to describe the act of eating both sides of the fish (i.e. vaginal as well as anal penetration)
*Kinyume cha maubile*	Against/Opposite of creation	The perception that the anus is meant for excretion and not sex and act of inserting the penis through the anus was considered going against creation
*Nipe mambo leo*	Give me an experience or something new	Could refer to adventure

In a discussion with women from the general population, they reported:

This practice is confidential. It is not as public as HIV, so people do not talk about it openly, [IDI, female, general population, 43 years).

The names of cellular phone companies were the commonest terms used by most participants in the general and key population. FSWs reported that while *vodacom* may refer to vagina, other terminologies like ‘*zain’* and ‘*tigo’* referred to the anus, which in most cases was linked to anal sex. Among the three cellular phone company names *'tigo'* was the commonest terminology used to describe HAS*:*

I think they call it [HAS] *tigo* to reduce the sharpness of the words…But also I think the *tigo* emblem is circular, it is like a zero, that is why they relate the anus with it and that is why they decided to call the act *tigo*, [IDI, Fishermen, 15-24 yrs].

The terms most women used to describe HAS were distinct from those men used. Majority of women described HAS using terms that could be considered discrete (e.g. *kinyume na maumbile* [opposite of creation]). Some participants described the use of metaphors to describe HAS as implying that the practice is frowned upon by the community. General population participants talked about this in the following:

These names are used because this thing is not good. They use these words so as to hide it from people. For example, I can say a certain person is with *tigo*, and you can’t know what I mean until someone tells you… “this person is fucked in the anus”, [FGD, females aged 24 -49 years, general population].

Although most men used metaphors that could be considered crude to describe HAS while with their peers, they were aware of the need to use more respectful language when negotiating HAS with their sexual partners. An example of crude terms men used while with their peers were ‘*kumtia nyuma*’ [fuck through the anus], and ‘*kutomba kwenye mkundu*’ [to fuck in the anus]. Truck drivers described their use of metaphors as follows:

It is in the course of trying to reduce the sharpness in the word *kufira* that someone uses that. It is like saying ‘to fuck’… You have to tell a woman in a descent manner… If you are a clever person, you can’t just tell her let’s go and fuck each other… Therefore, in the same way, those *wafiraji* thought ‘we should reduce the sharpness of this word, ‘*kufira’*, we should call it ‘*tigo’* [IDI, Truck driver, 30 yrs].

Almost all women reported that it was shameful disclosing to others that one had ever engaged in HAS. A woman who reported had previously engaged in HAS said:

Six months ago, my partner enticed me to have HAS. I was seriously hurt but did not tell anyone … Not even my mother. I lied that I was suffering from something else. You know, engaging in HAS is shameful …. You cannot disclose it to your parents, or any members of the family or even friends… You are the first person I have shared that secret, [IDI, female, general population, 25 years].

#### “Other” discourse

This discourse involves participants perception that it was distant others who engaged in HAS. Most participants talked about HAS as an alien practice that originated outside their communities. Key population participants other than FSW gave examples of those likely to engage in HAS, including Arabs, westerners/foreigners, mobile populations and those living along the coastal areas (perceived to have Arabic influence). A good example of ‘Other’ discourse were the accounts by most fishermen about those likely to engage in HAS as those working in bars, Muslim women, school girls, very poor women, women who engage in transactional sex, those living in urban settings with vibrant economies and the Westerners (“white people”). Within the same group, they admitted that HAS is also practiced by males from the fishing communities, but that this had been introduced to them by women who hanged around the fishing communities (e.g. lake shores and fishing camps) with a hope of having sex with fishermen in exchange for money. Truck drivers mentioned FSWs as the group most likely to engage in HAs:

This type of sexual practice is common in this population … You may take a prostitute and be completely ready for that (anal sex). When you tell a woman, “I will have sex with you through the anus”, that woman will tell you, “for anal sex you will need to pay me this much” you see?, [IDI, Truck driver, 25 years].

Similarly, general population groups gave examples of ‘Others’ likely to engage in HAS, including youth, unmarried people, wealthy men, FSWs and Arabs:

…Arab girls… before marriage have a tendency to practice anal sex so that they may preserve their virginity. Their intention is to be found virgins when they marry, [FGD, females, general population, 25-49 years].

#### Acceptability/trendiness discourse

The acceptability/trendiness discourses was common among young women and adult men. Within this discourse, participants described HAS as something trendy and increasingly gaining acceptability in their communities. Majority of young women working in FRFs and women from the general population were of the view that their peers engaged in HAS due to its trendiness as vaginal sex was perceived as old-fashioned. In a FGD with young women working in FRFs:

R1: They say oldies, the whole deal nowadays is at the back [anal sex], and at the front [vagina] they say oldies, I mean, it was once popular but that is no longer the case

R2: They say the front [vagina] has no flavour

R3: I mean the front is oldies, I mean it was used in the past but currently, they use the back [anus]

R4: Having it through the back [anus] is the trend currently, [FGD, females, FRFs, 15-24 years].

In a second FGD with young women from the general population:

For example, for us women, we sometimes want changes and this is what we call modern love, [FGD, females, general population, 15-24 years].

Participants referred to the differences in outlook about the practice as resulting from modernity and this was relatively common in young people than the older generations. FSWs contrasted the way HAS is talked about currently compared to the past in the following:

Honestly, engagement in HAS was kept a secret in the past … No one would reveal this secret to other people. However, the situation has changed nowadays … It [HAS] is openly discussed and no one is ashamed of engaging in HAS … It [HAS] is becoming more acceptable, just like vaginal sex, [IDI, FSW, 20 years].

Most young women and fishermen reported that some of their peers talked about HAS with pride, encouraging others to try the practice. A woman working in one FRF reported:

They talk about it while praising it [HAS], others are happy about it [talk]..I mean they see it as something important…another one says, “If I am with a man and he does not do that to me, I mean, I do not feel it [pleasure], [IDI, female, FRFs, 15-24 years].

One fisherman said:

You find that a young man has gone with a woman [had sex], when he comes back, he says “yesterday, I turned around my girlfriend a lot”… and for us we understand that to turn her is to have anal sex, [IDI, Fishermen, 25-49 years].

Most participants blamed globalisation as driving the trendiness discourse through media (pornographic movies) because many people accessed pornography through mobile phones:

I once got one man and when we were in [the room] next to each other, he opened his mobile phone and told me to select a style I liked. I told him that I didn’t want any style but he went ahead to show me a *chuma mboga* style (penetration through the anus while one is bending]…We get sensitized (about anal sex) through phones, [FGD, FRFs, 15 -24 years].

The trendiness of HAS was further supported by the belief that men who engaged in HAS were benefiting more than those who engaged in vaginal sex. A man from the general population talked about his experience with HAS after watching pornography in the following excerpt:

I saw from the video and felt that people were benefiting a lot and that when I was having vaginal sex with my wife, my wife was not groaning [sign of pleasure] then I thought that may be in the anus she would groan…So, I decided to have it in the anus. For sure it is sweet [pleasurable], [IDI, male, general population, 27 years].

#### Materiality discourse

The materiality discourse describes participants’ accounts of the economic benefits of HAS. Most of the females described HAS as a practice that is more profitable than vaginal sex while on the part of men they talked about HAS as something special and worth paying for. Men talked about males using HAS to punish women whom they had spend (through sexual exchange) with their hard earned money. Men described engaging in HAS as value for one’s money

R1: You know for us who work in the lake, you can stay there for even a month. When you return [to the shore], you want to use your money to relax. You have let’s say TShs 20,000 ($13) or TShs 30,000 ($20) and a woman asks for TShs 10,000 ($6.50). You enter [room] and start chatting, then it reaches a point of having sex, “my *mshikaji* [causal partner] can I have a certain amount of money”

R2: you decide I want to discipline her, you start working on her, you fuck her at the front, in the vagina, once done with the vagina, you turn her at the back [anus], [FGD, fishermen, 25-49 years].

Majority of young women from the general population and those working in FRFs perceived HAS as being widespread in their communities because women believed that HAS earns them more money in a short time period. Women working in FRFs especially those at alcohol selling venues talked about men wanting value for their money in the following:

He tells you, “are you ready for me to turn you around [have anal sex], then I will give you a large amount of money, even Tshs 200,000 ($133) so that you can give it to me at the back [anus], [FGD, FRFs, 15-24 years].

Young women from the general population said:

May be I should say that nowadays we go for value for money…Someone knows that if I do anal sex, I get much more money. Therefore, that behaviour is widespread…when other people hear this they say, “oh, in the anus you get much more money”, [FGD, females, general population, 15-24 years].

#### Masculinity discourse

This discourse relates to how males described engaging in HAS as an act of manhood: hence not wanting to say no to partners who initiated the act through their bodily movements during sex. Usually, it is men who were expected to initiate sex and make a decision on their preferred sexual act including HAS. Men talked about some women liking the practice but feared to directly tell their male partners that they preferred HAS than vaginal sex. Hence, men had to read the actions of women during a sexual encounter and act accordingly when a woman showed interest:

When you are having sex you do it in a normal way [vaginal sex], however,…When you are about to reach climax she pulls out the penis and inserts it in her anus, [IDI, male, general population, 20 years].

Participants talked about HAS as being used by men to punish women who had concurrent sexual partnerships but also as a way of proving to the woman that they were more experienced in sexual matters than the woman’s previous male sexual partners:

He just does it in order not to appear backward/not modern… he wants to show that he is also experienced in those things, [FGD, fishermen, 15-24 years].

#### Public health discourse

The public health discourse relates to how participants perceived and described the health risks of HAS and protection during the practice.

##### Knowledge of the link between HAS and HIV/STIs

Knowledge regarding the link between HAS and HIV infection was relatively high among the fishermen and women working in FRFs compared to other study populations. Most were able to describe possible risks of HIV infection through anal sex. Younger women in the general as well as key populations reflected a better and correct understanding of the linkage between HAS and HIV/STI infection than adult women and men in general. A young woman working at a FRF compared the safety of vaginal versus anal sex:

In my view, the vagina is much safer because even experts say that if you are well prepared for sex [foreplay], you can have it without any bruises and in that way that prevents you from HIV infection, [IDI, FRF, 24 years].

Another young woman described the link between HAS and risk for HIV infection as follows:

The anus is tight and without mucus. Hence, there is intensive friction and abrasion as compared with the vagina which is wider and has mucus…One doesn’t get abrasions because the penis does not experience tightness. Actually at the back it is easy for a man to get abrasions because it is tighter and as a man forces his way through your anus, you must get abrasion making it easy to get HIV infection, [IDI, FRF, 24 years].

However, in the discussions with adult men in the general population, there were a few participants who thought that HAS was safer than vaginal sex:

In reality, anal sex has no association with STIs because, the STIs are usually associated with the vagina, you see…,[FGD, males, general population, 24-49 years].

##### Perceived risks of HAS

Across all study populations, a commonality existed on what was perceived as health risks associated with HAS. However, some differences were observed among men and women. The main distinction was that while women reported complications during delivery, enlargement of the anus, and faecal incontinence, men cited anal blockages, occurrence of cancer, urinary bladder blockages*,* and condoms sticking in the anal cavity.

Most women talked about HAS being linked with difficulties women experience during childbirth due to weakened anal muscles. FSWs talked about this in the following:

It has consequences. You get severe pains during delivery. Instead of delivering the child from the front (*vagina*) the baby is delivered from the back (*anus*). That has happened, [IDI, FSW, 30 years].

Majority of women linked faecal incontinence with weakened anal muscles. Women from the general population said:

When you do it (*anal sex*) is a must that you get problems because the faeces will be coming out uncontrollably. Because all the sites are open, when you laugh or do anything it is a must that the faeces will come out, [FGD, females, general population, 25-49 years].

Most fishermen believed that HAS was linked to urinary tract infections and bladder blockage:

If you get used to fucking at the back it has consequences. You can suffer in old age, You will have a blocked bladder. It gets blocked. There is dirt that blocks the urinary tract, [FGD, fishermen, 25-49 years].

Some young women from the general population described how other people talked about HAS causing sperms to accumulate in the anal:

You know, I have an adult sister… just recently when she was admitted of stomach problems … the doctor told us, “this woman’s anus is blocked with sperms and it is important that you pay so that we can unblock that”…They unblocked the anus and by removing all the dirty things, [FGD, females, general population, 15-24 years].

##### Use of protective devises during HAS (e.g. condoms and lubricants)

Almost all participants were well aware of the protective effect of condoms against HIV infection. However, condoms available on the market were thought to be only suitable for vaginal sex and not inappropriate for HAS. Some talked about beliefs that if condoms were used during HAS they were likely to burst due to the tightness of the anus and hence there was no need for them. One woman said:

Even when a man decides to use a condom during anal sex, still you will get infection… in the anus whatever you do, it may not work. It is a must that one will be infected, [DI, female, general population, 25 years,].

Both men and women talked about people using lubricants for the reasons of easing penetration and not to avoid diseases. Hence, lubricants were described as common during HAS than were condoms for HIV/STI prevention. Examples of substances that were used as lubricants across the study populations were: petroleum jelly, saliva, K-Y Jelly, vaginal fluids, cooking oils, soaps, lotion and honey. A female from the general population talked about her experience in the following:

The man usually applies the jelly on his penis and around the anus so that when he enters his penis does not get resistance because that place [anus] is dry, [IDI, female, general population, 35 years].

However, some women from the general population talked about lubricants being beneficial for men than women:

Even if he [man] will apply oil, it will help him to enter [his penis] easily but you [woman] will still get bruises and tear your anus, [FGD, females, general population, 24-49 years,].

## Discussion

This study delineates six discourses that were used by participants from the general population and selected key population groups to talk about HAS. The discourses were simultaneously available to participants across the populations although some discourses were more common in certain sub-groups than others. The discourses in which participants positioned themselves changed as several participants drew on different discourses at different stages of the interview and discussion. It has been noted that whatever the discourse through which people might understand their behaviour, different discourses could be drawn on in a conversation as socially appropriate [11,13]. Therefore, when negotiating a sexual encounter, men may use different discourses from those they might use when discussing HAS with fellow men. Similarly, young women may use the trendiness/acceptability discourse when talking about HAS with peers but may align more with the public health discourse when with other audiences.

The discourse most prevalent among young women was the trendiness/acceptability of HAS discourse, while among men was the masculinity discourse. The materiality discourse was prevalent among women of all age groups regardless of the study population, while the ‘other’ and public health discourses were similarly discussed among both men and women across the different populations.

The terms used to describe HAS reflected the contexts within which participants lived. The symbols used by the cellular telephone companies in Tanzania seemed to influence the selection of metaphors for the practice. *Tigo*, *vodacom* and *Zain* telephone companies have a national reach and are represented by a circular shape. Drawing metaphors of HAS from names and symbols/logos of telephone companies may attest to the influence of everyday situations to label behaviours, including more recent but widely expanding mobile technologies.

Even though participants talked about HAS with ease, on the contrary, terms used to describe the practice were intended to hide it from other people outside their group. The ease with which many participants talked about HAS may imply that the practice could be explored further in surveys since this is something people may talk about when asked to. The use of diverse terms may imply a need to include multiple terms to describe HAS when measuring the magnitude of the practice.

The ‘other’ discourse is an indication of the stigma that may be attached to the practice [4]. Hence, most participants reporting that other groups of people outside their category, practised HAS but not themselves. Moreover, some of the terms (*e.g. kufira, msenge*) indicated a link of HAS with MSM. Linking HAS with MSM and the perception that it is others unrelated to them who engaged in HAS may further stigmatise the practice and make it difficult to understand personal experiences. The stigmatization of certain behaviours limits access to sexual and reproductive health services [29-31] as people may feel that it is others who are at risk and not them. Such stigma has also been linked to under reporting of risky sexual behaviours [32,33] for fear of rebuke and discrimination. There is need for further research to explore the extent of this belief that it is others unrelated to them who engaged in certain high-risk sexual practices.

Gender was important in determining how participants talked about HAS. While men described HAS using crude terms, women were more careful in their selection of terms and overall description of the practice. This observation could reflect social norms around masculinity and femininity in these communities that may tolerate male expression of ideas [34,35].

Accounts of men using HAS to punish women whom they had exchanged sex with money or those who had concurrent sexual partners could be indicative of the existence of sexual assault in some of the relationships. Studies have linked sexual violence with increased risk of HIV [36-38] and hence accounts of men about using anal sex to punish women could increase their HIV risk. Further research needs to be done to explore the extent to which some of the anal sexual encounters may be considered assault and how sexual assault in relationships is associated with increased risk of HIV.

Young people’s descriptions of HAS may imply a non-conservative attitude about the practice compared to the older generations. Young women were in support of the practice as indicated in the way they talked about HAS as trendy. The perception of HAS as trendy could be an indication of the socio-economic changes happening in these communities and young women’s perception of modernity. Similar findings about young people adopting behaviours that they considered trendy in a desire to move away from what they considered old-fashioned behaviours has been noted in other studies [34,39,40]. Although this discourse encourages HAS among young women, it offers important avenues to channel interventions aimed at reducing risks related to HAS. For instance, interventions, could consider ways of incorporating messages on condom use as trendy and acceptable for HAS.

The materiality discourse is a key driver for HAS among women. Women’s desire to earn as much money as possible in a short while, thus benefiting more financially by engaging in HAS than vaginal sex, is worrying. Similar findings have been noted in another study in Tanzania [21]. Interventions could take advantage of the negotiations for money during HAS to encourage negotiations for condoms as well.

It is apparent in the public health discourse that most participants were aware of the risks of HAS and its link to HIV and other STIs. However, this awareness did not discourage them from having unprotected HAS. As noted elsewhere, knowing the risk does not necessarily translate into adopting safer sex behaviours [41]. Participants discussed the type of condoms currently available on the market as not suitable for HAS and hence, using them during HAS was considered not effective against HIV infections. Participant’s views may reflect a genuine concern and a need for further work to explore condoms available in the country and increase the variety of affordable/subsidized condoms.

Structural factors, in particular economic circumstances and social norms influenced the discourses participants drew on. The positions men and women took in the discourses were shaped by norms on masculinity for men [33] while for both men and women by socio-economic contexts of their communities [42]. Women’s access to economic resources shaped the materiality discourses while the trendiness discourse was shaped by socio-economic changes. The masculinity and secrecy discourses may have been influenced by the social norms stipulating what is appropriate behaviour for men [33]. On the other hand, the public health discourse was shaped by HIV prevention interventions that participants previously had access to [43,44].

The key strengths of this formative study is the inclusion of general and key population groups, involving multiple sites in various parts of Tanzania and the utilisation of different methods allowing for the exploration of multiple dimensions of HAS. Despite these strengths, the following limitations of our study should be considered. First, it is difficult to establish whether the discourse used in the discussions to describe HAS behaviour was the same as that through which the behaviour was understood at the time of data collection. Second and more fundamental, is the difficulty in clarifying whether the discourse within which one positioned themselves prompted certain actions or whether having acted in a particular way, the person adopted a particular discourse through which to interpret their actions. Although our data does not suggest that relationship between discourse and practice, we are confident that practices could affect discourses that specific populations draw upon. Therefore, being part of a population (such as FSW, fishermen or truck drivers) or age group made certain discourse positions more available and legitimate than others. However, preference for particular discourses probably influenced selection of, and selection into friendship/occupational groups, which undermines any simple notion of causation.

## Conclusion

### Implications of these discourses for HIV

These discourses have implications for the magnitude of the practice, decisions on the practice of safer sex, and ultimately HIV prevention. The public health discourses points to the participants’ awareness of health risks of engaging in HAS, but that awareness may not translate into safer HAS because of other conflicting discourses such as trendiness and masculinity discourse. Since discourses are more than language, but organise meaning into action, it is important for interventions aimed at reducing risks related to HAS to consider competing discourses when addressing HAS risks.
